# Effectiveness of a Technology-Based Injury Prevention Program for Enhancing Mothers’ Knowledge of Child Safety: Protocol for a Randomized Controlled Trial

**DOI:** 10.2196/resprot.6216

**Published:** 2016-10-31

**Authors:** Chun Bong Chow, Wilfred Hing-Sang Wong, Wing Cheong Leung, Mary Hoi-Yin Tang, Ko Ling Chan, Calvin KL Or, Tim MH Li, Frederick Ka Wing Ho, Daniel Lo, Patrick Ip

**Affiliations:** ^1^ Department of Paediatrics and Adolescent Medicine The University of Hong Kong Hong Kong China; ^2^ Department of Obstetrics and Gynaecology, Kwong Wah Hospital Hong Kong China; ^3^ Department of Obstetrics and Gynaecology, The University of Hong Kong Hong Kong China; ^4^ Department of Social Work and Social Administration, The University of Hong Kong Hong Kong China; ^5^ Department of Industrial and Manufacturing Systems Engineering, The University of Hong Kong Hong Kong China

**Keywords:** child safety, injury prevention, technology-based intervention, anticipatory guidance, randomized controlled trial

## Abstract

**Background:**

Provision of anticipatory guidance for parents is recommended as an effective strategy to prevent injuries among young children. Technology-based anticipatory guidance has been suggested to reinforce the effectiveness of injury prevention and improve parents’ knowledge of child safety.

**Objective:**

This study aims to examine the effectiveness of a technology-based injury prevention program with parental anticipatory guidance for enhancing mothers’ knowledge of child safety.

**Methods:**

In this randomized controlled trial, 308 mothers will be recruited from the antenatal clinics and postnatal wards of two major public hospitals in Hong Kong. Participating mothers will be randomly assigned into intervention and control groups. Mothers in the intervention group will be given free access to a technology-based injury prevention program with anticipatory guidance, whereas mothers in the control group will be given a relevant booklet on parenting. The injury prevention program, available as a website or on a mobile app, includes behavioral components based on the Theory of Planned Behavior. The primary outcome measure will be the change in the mother’s knowledge of child safety. The secondary outcome measures will be age-appropriate domestic safety knowledge, attitudes, intentions, perceived behavioral control, and self-reported behavior related to home safety practice. We will also determine dose-response relationships between the outcome measures and the website and mobile app usage.

**Results:**

Enrolment of participants will begin in October 2016. Results are expected by June 2018.

**Conclusions:**

Parents will be able to easily access the domestic injury prevention website to find information regarding child injury prevention. It is anticipated that the technology-based intervention will help parents improve their knowledge of child safety and raise their awareness about the consequences of domestic injuries and the importance of prevention.

**Trial Registration:**

Clinicaltrials.gov

Clinicaltrials.gov NCT02835768; http://clinicaltrials.gov/ct2/show/NCT02835768 (Archived by WebCite at http://www.webcitation/6lbXYM6b9)

## Introduction

Childhood injury is a major public health problem worldwide [1]. According to the World Health Organization, approximately 46 out of every 100,000 children under the age of 5 years die from unintentional injuries [2]. Childhood injury is a significant health problem in Hong Kong. Between 2010 and 2012, more than 740,000 injury cases were reported in children aged 0 to 19 attending Accident and Emergency Departments in Hong Kong [3]. Specifically, pediatric unintentional domestic injury was one of the top three injury types among children in Hong Kong, accounting for 38.67% (286,193/740,000) of injuries in the 0 to 19 age group [3]. In the 0 to 4 age group, domestic injury was the leading cause of injury, accounting for 64% of all injuries. Unlike intentional injury caused by domestic violence, pediatric unintentional domestic injury is associated with the lack of available living space and safety measures, parents’ poor perception of injury risk, and children’s injury risk behaviors.

Parents are the primary caregivers and role models for their children’s behavior, and they should be given guidance regarding domestic injury prevention and how to modify the home to be a safe environment [4,5]. Although health care professionals are perceived as a credible provider of injury prevention information [6,7], parents will need to play an influential role in reducing their children’s exposure to injury risk by adopting better childcare practices and using appropriate child safety devices in the home [8,9]. Anticipatory guidance provided to parents during pediatric medical care is an effective strategy to prevent injuries and promote child health [10]. Anticipatory guidance consists of useful information for parents and families about their children’s development, and practical information on how to promote this development and adopt good practices [11]. Anticipatory guidance can be delivered in line with developmental milestones of a child [12]. Previous studies on the effectiveness of anticipatory guidance indicated that parent and child behavior outcomes were associated with reduced injuries and improved development during infancy and early childhood [7,13,14].

Technology-based interventions with anticipatory guidance information can be accessed anonymously, anytime, and anywhere. These systems can provide individualized and tailored interfaces enriched with interactive elements [15], and such interventions could be a useful approach for the prevention of domestic injury. Recent studies suggest that technology-based interventions can reinforce the effectiveness of injury prevention, and improve parents’ knowledge of child safety [16-18]. A systematic review of technology-based interventions for unintentional injury prevention highlighted 10 parenting interventions for child safety; all interventions were demonstrated to improve parents’ injury prevention behaviors [19]. For instance, a study by Van Beelen et al revealed that a Web-based intervention increased parents’ behavior toward child safety more than a standard leaflet related to scheduled childcare counseling [20]. Only two interventions evaluated changes in safety knowledge, and both showed positive changes [19]. A study by Gielen et al reported that an online intervention had a positive effect on enhancing safety knowledge [21]. Furthermore, the systematic review found only two out of 44 technology-based interventions were available as mobile apps [19]. Mobile apps for injury prevention intervention should be developed and thoroughly evaluated.

In this study, we propose to develop a new technology-based intervention that includes parental anticipatory guidance related to child injury prevention that is accessible on the web and as a mobile app. We will examine the effectiveness of the intervention in enhancing mothers’ knowledge of child safety. Using our new technology-based approach, we aim to motivate mothers to learn about pediatric unintentional domestic injury prevention to improve their knowledge of child safety, attitudes, and perceived behavioral control toward home safety practice. Website usage will be evaluated in terms of its reach and engagement statistics. User acceptance evaluations will be conducted at the end of the intervention period to collect feedback on the website interface.

## Methods

### Study Design

**Figure 1:**
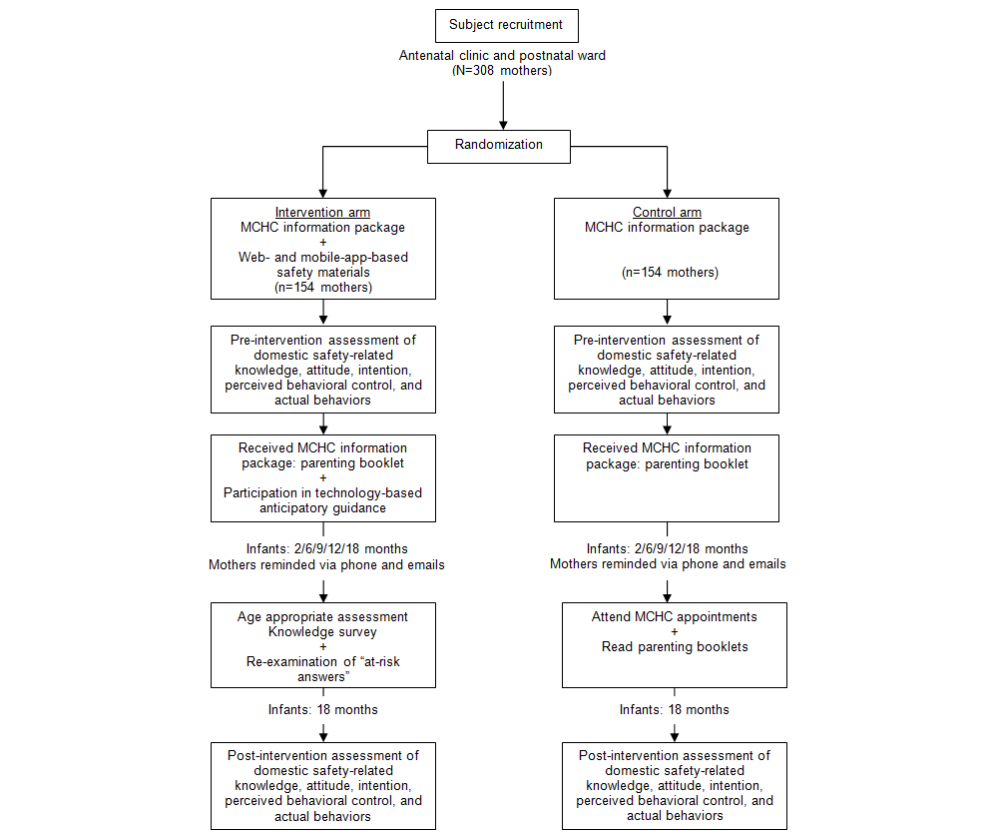
Flow diagram of study design.

This study will be a randomized controlled trial of the effectiveness of a technology-based injury prevention program for enhancing mothers’ knowledge of child safety (Figure 1). Mothers will be recruited from the antenatal clinics and postnatal wards of two major public hospitals in Hong Kong: Kwong Wah Hospital and Queen Mary Hospital. Between October, 2016 and January, 2017, nurses will identify potential participants. An experienced research assistant will provide the participants with an information sheet explaining the study, along with a consent form. The inclusion criteria will include mothers attending the antenatal clinics or staying in the postnatal wards at Kwong Wah Hospital or Queen Mary Hospital. The exclusion criteria will include subjects unable to read Chinese, and those without Internet access.

Participants will be randomly allocated into the intervention and control groups in a 1:1 ratio. All participants will be given an information pack consisting of a parenting booklet from the Maternal and Child Health Centers (MCHC). This booklet is publicly available and provides brief and general home safety tips for mothers with children under 3 years of age. The intervention group will also be given an additional leaflet containing a brief introduction to the Internet-based domestic safety platform, and instructions on how to access the website. Mothers can easily access this practical online parenting resource through the website or a mobile app. The online resource contains information on various safety topics and educational materials, including general and age-appropriate injury prevention measures suitable for mothers with infants and children between 2 and 18 months of age. Participants in the intervention group will be periodically contacted by telephone and electronic messaging to encourage them to access and engage with the domestic safety website. The research team will provide technical support to all participants who encounter problems accessing the intervention website or mobile app. Apart from technical support, no other intervention will be provided to those accessing the website or mobile app by any therapist, nurse, care provider, or physician. The control group will receive only the MCHC parenting booklet.

All consenting participants will be asked to complete a general safety questionnaire at the antenatal clinic or postnatal ward of the two hospitals. Age-appropriate safety knowledge, attitudes, intentions, perceived behavioral control, and self-reported behavior related to home safety practice will be assessed at specific child developmental ages at 2, 6, 9, 12, and 18 months. Participants in the intervention group will be given immediate feedback on their specific injury prevention behaviors prompted by the *at-risk* (incorrect) answers obtained from their completed questionnaire. This online feedback counseling approach aims to educate mothers and enhance their awareness related to domestic injury prevention. At the end of the counseling session, each intervention participant will be provided with age-appropriate safety information. Participants’ general safety knowledge will be reassessed when their children reach the age of 18 months. Intervention participants will be reminded by telephone and email to complete the age-appropriate safety questionnaires at each developmental stage, and the final general safety questionnaire. Control group participants will be contacted using the same schedule, to remind them to read the booklet and to complete the questionnaires.

This study is registered with Clinicaltrials.gov (Identifier: NCT02835768) and has been approved by the Institutional Review Board of Hong Kong University and Hospital Authority Hong Kong West Cluster (Reference number: UW 15-465). All participants will provide written informed consent.

### Sample Size Calculation

With reference to a computer-based parenting intervention for child injury prevention that reported a small to medium effect (d=0.42) for knowledge change [21], 92 subjects will be needed for this intervention study to detect an effect with 80% power and a 5% significance level. Assuming a 40% attrition rate between preevaluation and postevaluation, at least 154 participants should be recruited to each of the intervention and control groups.

### Randomization and Masking

Participants will be randomly assigned to the intervention group or the control group by stratified randomization within each hospital, using random numbers generated using R Statistical Software. Each participant will receive a sealed opaque envelope assigning them to either the intervention (website and mobile app access) or the control (no website or mobile app access) groups. The participant recruitment and randomization process will be independently carried out by different research assistants. Outcome assessors will be blinded to the allocation of participants in each group.

### Technology-Based Anticipatory Guidance

The technology-based intervention website will be developed using the existing injury prevention website designed by the Hong Kong Childhood Injury Prevention and Research Association (CIPRA), which provides informational, educational, and motivational support to parents [22]. The existing CIPRA website contains assorted safety information (not limited to domestic safety) and online games to deliver safety messages. The CIPRA website includes two key topics on (1) types of domestic injury, and (2) preventing a range of domestic injuries. In this study, the technology-based website used by the intervention group will be based on the CIPRA website, but with limited access to only the domestic safety and domestic injury prevention topics. Topics related to domestic injury will be temporarily suspended from the main CIPRA website during the study period. A mobile app will also be developed in conjunction with the website platform to facilitate participants’ acquisition of knowledge regarding child-related domestic injury prevention. The contents of the intervention website and mobile app will be the same.

The Injury Prevention Program (TIPP), an intervention website introduced by the American Academy of Pediatrics [23], will be the basis for the core strategy of our domestic injury prevention intervention. A review of the literature on childhood injury prevention demonstrated that TIPP was effective in improving safety knowledge and home safety practices [24]. Our intervention program will provide anticipatory guidance via the general domestic safety tips from the CIPRA website and TIPP. These combined resources will provide enriched domestic injury prevention information (*Home Safety Tips*) which will be divided into five stages according to the safety issues relevant to infants/young children in various age groups (Table 1). The intervention platform is intended to provide mothers with domestic injury prevention information specific to their children’s age, and important injury prevention information and messages will be age-appropriate for each developmental stage. Mothers will complete a corresponding safety survey at each developmental stage that their children reach. Safety counseling topics include common injury types such as falls, drowning, burns, and poisoning, but not unintentional firearm injury. Firearms, guns, and other weapons are prohibited by law in Hong Kong, so it is unlikely that children will be injured in such a way.

**Table 1:** Age-appropriate safety counseling topics adopted and modified based on The Injury Prevention Program (TIPP).

Age	Safety counseling topics
1.5 to 2 months	
	Falls
	Scalds
	Sleeping safety
	Suffocation
	Drowning
6 months	
	Falls
	Burns
	Medicine poisoning
	Drowning
	Toy hazards
9 months	
	Concussion
	Drowning
	Driving hazards
	Finger pinching
12 months	
	Poisoning
	Falls
	Sunburn
	Driving hazards
18 months	
	Poisoning
	Falls
	Sunburn
	Driving hazards

The value of using a behavioral approach to design an injury prevention program is widely recognized by researchers [24,25]. The Theory of Planned Behavior is a model that links beliefs and behavior to predict people’s intention to perform actual behaviors [26]. This intention comprises three important elements: attitude, subjective norms, and perceived behavioral control. Attitudes are derived from a person’s perceptions about the consequences of behavior and the importance of these consequences to the individual. Subjective norms are derived from one’s perception of beliefs about significant others’ preferences and the individual’s motivation to comply with their wishes. Perceived behavioral control is derived from a person’s perception of the difficulty to conduct the behavior. Adriaensens et al reported that an online intervention was able to deliver a positive effect on knowledge, attitudes, intentions, and actual injury-preventive behavior [27]. Therefore, besides being a knowledge hub, the intervention website and mobile app will incorporate additional feedback counseling, online consultations, a discussion forum, interactive games, and video demonstrations to improve participant’s attitudes, intentions, and perceived behavioral control toward home safety practices.

Mothers can use the discussion forum to exchange opinions or ideas related to domestic safety, such as the usefulness of safety gadgets, uncommon domestic injury hazards, and home safety emergency plans. Concerned mothers can also use the forum for online consultation by asking questions about childhood injury, and can even upload photos, which will then be answered by safety experts. However, in an emergency, participants should contact emergency services and attend their nearest accident and emergency department. The forum can serve as a practical learning platform and provide parents or caregivers with additional social value through online interaction. Online discussion forums have been shown to enhance social support and the feeling of parental efficacy, and thus can improve one’s attitudes and intentions to adopt safer practices [18].

Additional interactive elements such as video demonstrations, games, and quizzes will be added to the website and mobile app. Two interactive games have been designed to deliver key information related to childhood safety and injury prevention. In the first game (named *spot the difference*), players need to identify differences in potential home hazards between two pictures. Explanations will be provided when players spot the potential home hazard, such as the type of injury (eg, scald and poisoning) that the hazard would cause. In the second game (named *cue and action*), players need to select a particular prevention tool to stop the potential hazard that may cause domestic injury. These interactive games and video demonstrations would help the participant create mnemonics for injury prevention, and enhance one’s perceived behavioral control toward home safety practices. Safety practice demonstrations by health care providers who are role models and influencers have been reported to be useful in engaging mothers and reinforcing positive outcomes [6,7].

The website will contain comprehensive injury prevention information and useful links and telephone numbers. All information contained in the website and the app will be consolidated and updated regularly, taking into account the changing patterns arising from local injury profiles, caregivers’ responses, and the physical environment. Reference and guidance materials such as injury-related publications of evidence-based good practices, statistical data, and frequently asked questions will be made available on the website to reinforce users’ perceived behavior control toward domestic injury prevention. In addition, the injury profiles of the 18 districts in Hong Kong, with infographic summaries, will be uploaded to the website for reference [3].

### Technology

The existing CIPRA website will be redesigned to accommodate the intervention program in this study, and will be cohosted by CIPRA and the authors’ department. A mobile app will be made available for downloaded on Android phones and iPhones. The website will run on a Linux server located and managed under the authors’ department, and will have regular backups to prevent any accidental data loss.

### Measures

This study aims to increase mothers’ knowledge of child domestic safety, and to improve their attitudes, intentions, perceived behavioral control, and actual behavior related to home safety practice. The following five evaluation components will be included to demonstrate the effectiveness of the technology-based intervention: general safety knowledge, age-appropriate safety questionnaire, injury prevention behavior checklist, website and mobile app usage statistics, and website and mobile app user acceptance.

#### General Safety Knowledge

This component aims to evaluate changes in mothers’ general knowledge related to domestic injury prevention [28,29]. The items in the questionnaire are in a statement format and cover two key topics: (1) common childhood domestic injuries, and (2) safety precautions (see Multimedia Appendix 1).

#### Age-Appropriate Safety Questionnaire

This component examines age-appropriate domestic safety knowledge, attitudes, intentions, and perceived behavioral control toward home safety practice (Table 1) [30-32]. The multiple-choice questions in the questionnaire are categorized into two sections: (1) age-specific questions, and (2) assessment questions that were answered incorrectly in the previous stage by the participant (see Multimedia Appendix 2). This targeted component is designed to reinforce safety concepts and sustain knowledge enhancement.

#### Injury Prevention Behavior Checklist

This component examines different age-appropriate injury prevention behaviors. The injury prevention behavior checklist serves as a behavioral measure for: (1) identifying hazards, and (2) implementing safety measures in a home environment (see Multimedia Appendix 3).

#### Website and Mobile App Usage Statistics

Participants will be required to register online as a *user* to access the website and the mobile app. By logging into the system, we will be able to record all actions taken by each user, including login time, duration of website usage, and what information was accessed. The captured data can be further analyzed and used for the purpose of incentive reimbursement.

#### Website and Mobile App User Acceptance

Towards the end of the intervention period, subjects will be reminded to complete a user acceptance evaluation (see Multimedia Appendix 4). The practicability of the technology-based domestic injury prevention website and mobile app will be evaluated in terms of its layout, structure, usability, readability, accessibility, and ease of navigation [33].

### Incentives

To enhance participation and minimize attrition in the follow-up months, incentives will be given to mothers who continue to complete evaluations. Proposed incentives include safety gadgets and cash coupons (valued at Hong Kong $50; approximately US $6.40) from companies that promote child domestic safety.

### Data Analysis

Changes in participants’ knowledge of child safety will be studied by comparing their knowledge before and after completing the injury prevention program, and by comparing the participants’ knowledge of child safety in the intervention and control groups. Regression analysis will be used to examine the intervention effectiveness, adjusted for participant demographics (eg, gender, age, and socioeconomic status of the parent). Data will be analyzed by an intent-to-treat approach to address loss of any follow-up data. Independent sample t-tests will be used to examine the between-group differences in age-appropriate safety knowledge, attitudes, intentions, perceived behavioral control, and actual behavior related to home safety practice. Dose-response relationships between the outcome measures and the website and mobile app usage will be determined by correlation analysis. Website and mobile app usage data will be summarized based on the number of users and their time spent using these resources during the entire intervention period. A summary of the user acceptance evaluation will be compiled to improve and develop the website and mobile app.

## Results

This project was successfully funded by the Health Care and Promotion Fund (Project No. 08150345) of the Food and Health Bureau, Hong Kong government, in March, 2016 after a stringent external review process. Enrolment of participants will start in October, 2016 and results are expected by June, 2018.

It is anticipated that the technology-based injury prevention intervention will yield a small to medium effect in terms of mothers’ child safety knowledge enhancement. It is estimated that intervention participants will spend more time on the mobile app than on the website. Dose-response relationships between the outcome measures and the website and mobile app usage will be examined. It is expected that intervention participants will have better age-appropriate safety knowledge, attitudes, intentions, perceived behavioral control, and actual behavior related to home safety practice compared to the control group participants.

## Discussion

This study examines the effectiveness of a technology-based intervention designed to improve mothers’ awareness of the severity and consequences of domestic injuries. Mothers can enhance their knowledge of child safety by accessing the online age-appropriate information related to child health, parenting, and safety. This technology-based platform can help users find available resources, services, providers, and relevant contact information.

A discussion forum will be available on the new website and mobile app as a means to facilitate communication between mothers and professionals. Mothers can also benefit from technology-mediated social support and chat rooms, and exchange lay knowledge. Safety experts and pediatricians will be invited to provide advice, professional support, and counseling through the website and the app, which will strengthen online services and promote engagement. The website will help to mobilize community stakeholders to address child safety concerns and other aspects of child health, and will allow stakeholders to raise their own concerns. The website will empower parents to take responsibility for their children’s health and safety, and to adopt good health practices.

The Internet is becoming widely accepted as an effective, low-cost platform for the dissemination of health and safety information. In comparison to traditional media, information on the Internet can be updated instantly and inexpensively [34]. Electronic dissemination of information has a great deal of potential when compared to traditional media, especially when considering the speed of information propagation, because it has the ability to reach a large audience and connect with those who might otherwise be difficult to approach [35]. Technology-based interventions can also serve as platforms for viral campaigns, which involve sharing knowledge among information seekers. This easily accessible online safety platform (intervention website and mobile app) containing comprehensive up-to-date information on childhood safety will educate parents and caregivers, and encourage them to adopt safer practices. Findings from the study will contribute to good health practices, future research, and the development of user-friendly online information platforms. For example, the educational effort of CIPRA could be enhanced by combining other approaches, including legislative measures and infrastructure improvements, because a solely educational approach is limited in reach. Our main goal is to provide an effective online educational platform to be funded on an ongoing basis, which will be incorporated into the Maternal and Child Health Services under the Department of Health, and into postnatal clinics in public hospitals.

This study will be carried out in collaboration with CIPRA by sharing resources, exchanging best practices, and combining efforts towards sustainability. Furthermore, this Web-based intervention will demonstrate a sustainable health promotion strategy that mobilizes local resources with cross-sectoral collaborations to engage the community by building partnerships between private, public, and nongovernmental organizations. Upon completion of the evaluation, the information leaflet with details of the domestic safety website will be disseminated to relevant public, private, and nongovernmental organizations that promote child health, and particularly child safety. This freely accessible safety platform will allow organizations to reinforce awareness and promote domestic injury prevention among target populations.

Information on the platform can be revised regularly to ensure that users have access to high quality, up-to-date, and accurate information. All safety information contained on the website and mobile app will be shared with interested nongovernmental organizations, MCHC, and postnatal clinics. All information associated with the platform can also be linked across sectors, including the government, academic institutions, nongovernmental organizations, and private sectors in Hong Kong. The CIPRA safety information website and mobile app are expected to be easily accessible for all users, including parents, caregivers, school teachers, social workers, children, and nonacademic medical and allied health professionals. This platform will serve as an information hub for the community and a learning platform for all relevant stakeholders. When disseminating the findings from the intervention study within the community, we will also compare the intervention with other services by collecting feedback from different users, interviewing relevant stakeholders, and conducting ecological and econometric studies to assess the long-term social impact on the intervention. We will further improve the intervention platform to meet the needs of the community.

Anticipatory guidance for injury prevention should be provided to parents along with routine medical care, such as well-child visits for infants and children. An important advantage of this Internet-based intervention, apart from providing anticipatory guidance, is its potential to reach a wide audience, which can be exploited by other electronic health services that utilize community-wide interventions with intersectoral collaborations between public, private, and nongovernment organizations. The use of technology-based interventions is increasing, and can be considered a powerful mobilization strategy for engaging various user groups. In the future, the online platform can be adapted to deliver preventive pediatric health care information in other learning areas, such as growth monitoring, nutrition, and vaccinations. All of these important child health-related areas would contribute to the establishment of a child health portal that will help to promote the well-being and optimal development of children in Hong Kong.

## Appendix

Multimedia Appendix 1 General safety knowledge questionnaire.

Multimedia Appendix 2 Age-appropriate safety questionnaire.

Multimedia Appendix 3 Injury prevention behavior checklist.

Multimedia Appendix 4 Website and mobile app user acceptance questionnaire.

Multimedia Appendix 5 The peer-review report from the Health Care and Promotion Fund.

Multimedia Appendix 6 CONSORT-EHEALTH checklist (V1.6.1) [36].

## Abbreviations

CIPRA: The Hong Kong Childhood Injury Prevention and Research Association
MCHC: Maternal and Child Health Centers
TIPP: The Injury Prevention Program
