# Supplementary material for: Effectiveness of a Technology-Based Injury Prevention Program for Enhancing Mothers’ Knowledge of Child Safety: Protocol for a Randomized Controlled Trial
Source: JMIR Res Protoc. 2016 Oct 31;5(4):e205. doi: 10.2196/resprot.6216 (PMC5108924; doi:10.2196/resprot.6216)
Supplement: Multimedia Appendix 1 [file resprot_v5i4e205_app1.pdf]

The University of Hong Kong
香港大學兒童及青少年科學系
DEPARTMENT OF PAEDIATRICS AND ADOLESCENT MEDICINE
THE UNIVERSITY OF HONG KONG
Queen Mary Hospital
廣華醫院
Kwong Wah Hospital

General Safety Knowledge and Attitude Survey

A. Are you the **main caregiver** of your child?

Yes ☐ No☐

B. Do you know how to access the Internet?

Yes ☐ No☐

C. Have your children suffered an injury in the past three months

Yes ☐(Please answer [D]) No☐ (Please answer question 1)

D. What kind of injury have your children suffered in the past three months? :________________________

***Please put a □ at the most appropriate answer:**

1. To what extent do you think each of the following would help to keep your child from getting injured in an accident?

	**Not At All**	**Some**	**A Lot**
Car seat	☐	☐	☐
Cabinet lock	☐	☐	☐
Kitchen/toilet gate	☐	☐	☐
Fence with a locked gate around swimming pool	☐	☐	☐
Corner bumper	☐	☐	☐
Anti-slip mat	☐	☐	☐
Window frame (With lock)	☐	☐	☐
Safety plug	☐	☐	☐

Next you will be asked some questions about different types of safety measures that parents may or may not adopt. Each parent may choose differently. There are no right or wrong answers. For each question, please put a at the most appropriate answer.

2. What is the temperature setting on your hot water heater? (That is, what is the temperature of the water that comes out of your hot water faucet?)

☐ 48 degrees Celsius or 120 degrees Fahrenheit or less (skip to Question 3)

☐ Over 48 degrees Celsius or 120 degrees Fahrenheit but under 54 degrees Celsius or 130 degrees Fahrenheit

☐ More than 54 degrees Celsius or 130 degrees Fahrenheit

☐ I don’t know

[IF I DON’T KNOW]

2a. Have you ever thought about checking the temperature of the bathing water?

☐ No, I haven’t really thought about it

☐ I’ve thought about doing it

☐ I plan to check it soon

[If over 48 degrees Celsius or 120 degrees Fahrenheit]

2b. Have you thought about changing the hot water heater setting to reduce the temperature to 48 degrees Celsius or 120 degrees Fahrenheit or less?

☐ No, I haven’t really thought about changing the temperature.

☐ I’ve thought about doing it

☐ I plan to change it soon.

☐ I don’t have access to the hot water heater.

3. To the best of your knowledge, has anyone in your family ever held your child, even once or twice, while cooking or holding a hot liquid? (For example, holding a coffee cup or pan of hot water?)

☐ Yes

☐ No

4. Do you have a kitchen gate put in front of the entrance of your kitchen that your child cannot get to?

☐ Yes

☐ No

[IF NO]

4a. Have you thought about putting a kitchen gate in your home?

☐NO,I haven’t really thought about using them

☐I’ve thought about using them

☐I’m going to get one soon

[IF YES]

4b. How often do you keep your kitchen gate closed?

☐ Rarely or never

☐ Some of the time

☐ Most of the time

☐ Always

5. Does your child use a walker with wheels that roll?

☐ Yes

☐No

[IF NO]

5a. Have you thought about getting a walker

☐ No, I’m not going to get one

☐ I’ve thought about getting one

☐ I’m going to get one

6. In your home, where are cleaning products kept?

☐ Out on top counter or tables

☐ In cabinets below waist level

☐ In cabinets above waist level

[IF IN CABINETS]

6a. Are there cabinet locks on these cabinets?

☐ Yes

☐ No

[IF NO]

6b. Have you thought about putting lock on these cabinets?

☐ No, I've not really thought about using them

☐ I've thought about using them

☐ I plan to put them on soon

6c. In your home, where are medicines kept?

☐ Out on top of counter or tables

☐ In cabinets below waist level

☐ In cabinets above waist level

[IF IN CABINETS]

6d. Are there cabinet locks on these cabinets?

☐ Yes

☐ No

[IF NO]

6e. Have you thought about putting locks on these cabinets?

☐ No, I've not really thought about using them

☐ I've thought about using them

☐ I'm plan to put them on soon

7. Does your child have his/her own car seat?

☐ Yes

☐ No

7a. When your car is being ridden, how often is your child in a car seat?

☐ Rarely or never

☐ Some of the time

☐ Most of the time

☐ Always

[IF RARELY OR NEVER]

7b. Have you thought about using a car seat?

☐ No, I haven't thought about using a car seat

☐ I've thought about it

☐ I'm going to use one soon

[IF SOMETIMES]

7c. Have you thought about using a car seat more often?

☐ No, I haven't really thought about it

☐ I've thought about it

☐ I'm going to use it more often

[IF SOMETIMES, MOST OF THE TIME OR ALWAYS]

7d. Does the car seat face the front or back of the car?

☐Front facing (my child looks towards the front of the car)

☐Rear facing (my child looks towards the back of the car)

7e. Where in the car is your child usually seated?

☐ In the front seat

☐ In the back seat

[IF ALWAYS]

7f. Do you ever take your child out of the car seat for a short time, like to feed he/she or if he/she is fussy?

☐ Yes

☐ No

8. Has anyone in your family ever left your child alone in the area of the tub of water, even for a moment? (With or without a bathtub ring)

☐ Yes

☐ No

Next, you will be asked some questions about injuries to children–i.e.children getting hurt in an accident. For each question, pick the answer that is closestto what you think.

How would you comment each of the followings?

9. How much concern would you have on the phenomenon of domestic childhood injuries?

Not concerned at all 1 2 3 4 5 Very Concerned

10. How would you rate this statement "Domestic injury is preventable"?

Totally disagree 1 2 3 4 5 Totally Agree

11. For me, to get my child away from injuries is:

Totally unimportant 1 2 3 4 5 Totally important

12. For me, to create a safety living environment for my child is…………………………

Totally unimportant 1 2 3 4 5 Totally important

13. For me to practice domestic injury prevention behavior is………..

Totally invaluable 1 2 3 4 5 Totally valuable

14. Given you are actually adopting safety practice as recommended, what would you expect its effect on your child's future occurrence of injuries?

No effect at all on injury 1 2 3 4 5 Immensely reducing the chance of injury

**15. Any people would significantly influence your domestic injury prevention action?**							
My child	Totally unlikely	1	2	3	4	5	Totally likely
Family member:_________	Totally unlikely	1	2	3	4	5	Totally likely

Friend	Totally unlikely	1	2	3	4	5	Totally likely
Others:________________	Totally unlikely	1	2	3	4	5	Totally likely

16. For me to carry out domestic injury prevention behavior on a regular basis is…………

Totally difficult 1 2 3 4 5 Totally easy

17. How certain you consider able to create a safety environment for your child?

Totally unsure 1 2 3 4 5 Totally certain

18. To what extent does the physical environment of your household affect your effort on injury prevention, For Example: The size of flat has no or little support for my effort on injury prevention practice?

Totally likely 1 2 3 4 5 Totally unlikely

19. To what extent does the psychological environment of your household affect your effort on injury prevention, For Example: My spouse at home showed no or little support for my effort on injury prevention practice, I don't have time to implement the recommended injury prevention practices or modifications?

Totally impeding 1 2 3 4 5 Totally facilitating

20. Given you have been equipped with professional advice on how to prevent domestic injury and injury prevention devices (e.g. corner bumper for furniture), how would you conceive the idea about carrying out injury prevention practice by yourself?

Not a chance 1 2 3 4 5 Totally likely

21. How easy or difficult would it be for you to do each of the following?

	Easy	Somewhat Easy	Somewhat Difficult	Difficult
Put in the car seat facing the correct direction rather than the incorrect direction?	☐	☐	☐	☐
Put the car seat in the back seat?	☐	☐	☐	☐
Always keep your child in the car seat when the car is moving?	☐	☐	☐	☐
Check the bathing temperature before putting your child into the bathtub?	☐	☐	☐	☐
Never hold your child while cooking or holding a hot liquid?	☐	☐	☐	☐
Have a kitchen gate put up at all the times?	☐	☐	☐	☐
Never let your child use a walker?	☐	☐	☐	☐
Keep medicines above waist level in locked cabinets?	☐	☐	☐	☐
Never leave your child alone in a tub of water?	☐	☐	☐	☐
Never let your child exposed under the sun without any protection?	☐	☐	☐	☐
Use anti-slip mats?	☐	☐	☐	☐
Corner bumpers used for furniture?	☐	☐	☐	☐
Use safety plugs?	☐	☐	☐	☐
Use window frame(with lock)	☐	☐	☐	☐

22. I plan to carry out domestic injury prevention behavior in our home on a regular basis.

Totally unlikely 1 2 3 4 5 Totally likely

23. I will make an effort to have the household hazard check up on a regular basis.

I definitely will not	1	2	3	4	5	I definitely will

**24. In the past three months, have you implemented the injury prevention behavior below?**		
	Yes	No
Safety car seat in the back seat?	☐	☐
Always keep your child in the car seat when the car is moving?	☐	☐
Check the bathing temperature before putting your child into the bathtub?	☐	☐
Never hold your child while cooking or holding a hot liquid?	☐	☐
Have a kitchen gate put up at all the times?	☐	☐
Never let your child use a walker?	☐	☐
Keep medicines above waist level in locked cabinets?	☐	☐
Never leave your child alone in a tub of water?	☐	☐
Never let your child exposed under the sun without any protection?	☐	☐
Use anti-slip mats?	☐	☐
Corner bumpers used for furniture?	☐	☐
Use safety plugs?	☐	☐
Use window frame(with lock)	☐	☐

<u>Interviewee information:</u>

Q1. Gender

1. ☐ Male 2. ☐ Female

Q2. Age

☐Below 25 year-old☐ 26-30 year-old☐ 31-35 year-old ☐36-40 year-old ☐40 year-old or more

Q3. Age of your child: ____________________________days/week/months*

(*Please **Circle** the applicable option)

Q4. Educational level

1. ☐No formal education 2. ☐Primary school 3. ☐ Secondary school 4. ☐Post-secondary education

Q5. The relationship between your child and you

1. ☐ Father
2. ☐ Mother
3. ☐ Grandparent
4. ☐ Relative (Please specify: ____________________)
5. ☐ Living together but without any relationship
6. ☐ Neighbor
7. ☐ Other (Please specify: ____________________)

Q6. Family monthly income

1. ☐ Below HK$4,000
2. ☐ HK$4,000 – HK$7,999
3. ☐ HK$8,000 – HK$11,999
4. ☐ HK$12,000 – HK$15,999
5. ☐ HK$16,000 – HK$19,999
6. ☐ HK$20,000 – HK$29,999
7. ☐ HK$30,000 – HK$39,999
8. ☐ HK$40,000 – HK$49,999
9. ☐ HK$50,000 – HK$79,999
10. ☐ Over HK$80,000

Q7. Size of your home area

1. ☐Below 200ft 2. ☐200-400ft

3. ☐401 – 600ft 4. ☐601-800ft

5. ☐801-1,000ft 6. ☐1,001 – 1,500ft

7. ☐Over 1,500ft

Q8. Working status:

1. ☐ Employed – Full time
2. ☐ Employed – Part time
3. ☐ Non-engaged
4. ☐ Student
5. ☐ Retired
6. ☐ Housewife

Thank you for your participation
