# Supplementary material for: Effectiveness of a Technology-Based Injury Prevention Program for Enhancing Mothers’ Knowledge of Child Safety: Protocol for a Randomized Controlled Trial
Source: JMIR Res Protoc. 2016 Oct 31;5(4):e205. doi: 10.2196/resprot.6216 (PMC5108924; doi:10.2196/resprot.6216)
Supplement: Multimedia Appendix 2 [file resprot_v5i4e205_app2.pdf]

The University of Hong Kong
香港大學兒童及青少年科學系
DEPARTMENT OF PAEDIATRICS AND ADOLESCENT MEDICINE
THE UNIVERSITY OF HONG KONG
Queen Mary Hospital
廣華醫院
Kwong Wah Hospital

THE FIRST AND A HALF TO SECOND MONTHS OF AGE

THE SAFETY SURVEY

Please **Circle** the answer for each question.

1. Do you ever drink or carry hot liquids when holding your baby?	**Always**	**Sometimes**	**Never**
2. Does your child wear a pacifier or jewelry around his or his or her neck?	**Never**	**Occasionally**	**Frequently**
3. Do you keep plastic wrappers, plastic bags, and balloons away from your children?	**Always**	**Sometimes**	**Never**
4. Do you leave the baby alone in or near a tub, pail of water, or toilet, even for a brief moment?	**Never**	**Occasionally**	**Frequently**
5. Do you have a pool or hot tub where you live?	**Yes**	**No**	
6. Do you check the temperature of the bath water before bathing your child?	**Always**	**Sometimes**	**Never**
7. Do you check the temperature of the milk before feeding your child?	**Never**	**Sometimes**	**Always**
8. Does your child play with small objects such as beads or nuts?	**Frequently**	**Occasionally**	**Never**

9. Do you let your child sleep on a soft surface?	**Never**	**Occasionally**	**Frequently**
10. Do you get everything prepare to make sure you can stay with your child for bath time?	**Always**	**Sometimes**	**Never**
11. How do you place your baby to sleep?	**Sleep on his back**	**Prone**	**Sleep in one side**
12. Does your baby sleep on a large soft pillow?	**Always**	**Sometimes**	**Never**

The University of Hong Kong
香港大學兒童及青少年科學系
DEPARTMENT OF PAEDIATRICS AND ADOLESCENT MEDICINE
THE UNIVERSITY OF HONG KONG
Queen Mary Hospital
廣華醫院
Kwong Wah Hospital

THE SIXTH MONTH OF AGE

THE SAFETY SURVEY

Re-examination Question

Please **Circle** the answer for each question.

OccasionallyFrequently**1. Do you ever use oil-filled radiator or fan heater?**	**Always**	**Sometimes**	**Never**
**3. Does anyone in your home ever smoke?**Never<br>2. Do you keep electrical appliances and cords out of reach from your child?	**Frequently**	**Occasionally**	**Never**
4. Do you keep matches and cigarette lighters out of the reach from your child?	**Never**	**Sometimes**	**Always**
5. Do you have a plan for escape from the home in the event of a fire?	**Yes**	**No**	
6. Do you keep the handles of pots and pans on the stove out of the reach of your child?	**Never**	**Sometimes**	**Always**

7. Do you check your child's toys for safety hazards?	**Never**	**Sometimes**	**Always**
8. Do you keep your medicine out of reach and out of sight from your child?	**Always**	**Sometimes**	**Never**
9. Do you use safety locks or latches on drawers and cupboards?	**Never**	**Sometimes**	**Always**

The University of Hong Kong
香港大學兒童及青少年科學系
DEPARTMENT OF PAEDIATRICS AND ADOLESCENT MEDICINE
THE UNIVERSITY OF HONG KONG
Queen Mary Hospital
廣華醫院
Kwong Wah Hospital

THE NINTH MONTH OF AGE

THE SAFETY SURVEY

Re-examination Question

Please Circle the answer for each question.

1.Do you leave your child alone in the bathtub?	**Frequently**	**Occasionally**	**Never**
2. Do you take your child on a boat?	**Never**	**Occasionally**	**Frequently**
3. Do you have a pool or hot tub where you live?	**Yes**	**No**	
4. Do you allow your child to swim unsupervised?	**Frequently**	**Occasionally**	**Never**
5. Do you have a car safety seat in the car on every trip at all times?	**No**	**Yes**	**Not applicable**
6. Does your car have a passenger air bag?	**Yes**	**No**	**Not applicable**

7. Where do you place your child's car safety seat in the car?	**Front**	**Rear**	**Front or rear**	**Not applicable**
8. Do you shake your child whenever you are or not playing with him/her?	**Frequently**	**Occasionally**	**Never**	

The University of Hong Kong
香港大學兒童及青少年科學系
DEPARTMENT OF PAEDIATRICS AND ADOLESCENT MEDICINE
THE UNIVERSITY OF HONG KONG
Queen Mary Hospital
廣華醫院
Kwong Wah Hospital

THE TWELFTH & EIGHTEENTH MONTH OF AGE

THE SAFETY SURVEY

Re-examination Question

Please **Circle** the answer for each question.

1. Do you dispose old medicines?	**Always**	**Sometimes**	**Never**
2. Do you have safety caps on all bottles of medicine?	**Never**	**Sometimes**	**Always**
3. Does your child chew on paint chips or window sills?	**Yes**	**No**	
4. Do you put the crib side up whenever you leave your baby in the crib?	**Never**	**Sometimes**	**Always**
5. Do you have the baby alone on tables or beds, even for a brief moment?	**Frequently**	**Occasionally**	**Never**
6. Do you leave the baby alone?	**Never**	**Occasionally**	**Frequently**

7. Do you let your child under direct exposure of the sun without any protection over your child?	**Frequently**	**Occasionally**	**Never**	
8. Are your operable window guards in place?	**All windows**	**Some windows**	**None**	
9. How are your children restrained when they ride in a car?	**Car seat**	**Booster seat**	**Seat belt**	**None**
10. Do you leave your child alone in the car?	**Never**	**Occasionally**	**Frequently**	
11. Where do you arrange your child in the car?	**Front**	**Rear**	**Front or rear**	
12. Does your car have a passenger air bag?	**Yes**	**No**		
13. Do you lock the car doors before driving?	**Always**	**Sometimes**	**Never**	
14. Does your child play in the drive way or in or near the street?	**Frequently**	**Occasionally**	**Never**	
