# Supplementary material for: Effectiveness of a Technology-Based Injury Prevention Program for Enhancing Mothers’ Knowledge of Child Safety: Protocol for a Randomized Controlled Trial
Source: JMIR Res Protoc. 2016 Oct 31;5(4):e205. doi: 10.2196/resprot.6216 (PMC5108924; doi:10.2196/resprot.6216)
Supplement: Multimedia Appendix 3 [file resprot_v5i4e205_app3.pdf]

The first 2 months after birth

- Lock and put the crib side up whenever your baby is in the crib.
- Do not leave your child unattended on bed, diaper changing mat, table or couch
- Use non-slip mat in bathtub and bathroom.
- Fasten the seatbelt on the highchair or stroller.
- Do not allow your children to play in the bedroom or other places unattended.
- When a child is near or when you hold them, do not drink or carry any hot liquids simultaneously. Avoid using tablecloths and make sure to keep mugs, saucers and any hot liquids a reasonable distance from the edge of a surface.
- Make sure that the temperature of the water heater is not higher than 48 degrees Celsius or 120 degrees Fahrenheit. For bathing, add cold water into the tub first and then add hot water and mix it well. Examine the water temperature using your elbow.
- Before bottle-feeding or feeding any hot food, make sure to check the temperature of the milk and food to avoid scalding the children.
- Do not use microwave to prepare milk or food for your child.
- Let your child sleep on his back.
- Make sure that children sleep on a firm and reasonable size mattress.
- Do not place soft objects near the child when he sleeps (pillows, blankets, light soft duvets, pillows or stuffed toys and so on) and make sure the bedding isn't loose.
- Avoid the child from over-heating.
- Sleep with the children in the same room. Place the children's crib beside your bed and allow the children to sleep by themselves in the crib.
- Do not smoke.
- Place all small objects or food items away from the reach of young children.
- Dispose or store plastic bags properly.
- Tie up the curtain cords.
- Store the foldaway furniture such as tables and chairs properly.
- Do not take your child on board in a boat or ferry.

6 months after birth

- Lock and put the crib side up whenever your baby is in the crib.
- Do not leave your child unattended on bed, diaper changing mat, table or couch.
- Install window guards.
- Install gates around balconies.
- Use non-slip mat in bathtub and bathroom.

- Do not use walkers to avoid accidents.
- Fasten the seatbelt on the highchair or stroller.
- Do not allow your children to play in the bedroom or other places unattended.
- Place some barriers to keep the heaters out of reach from children.
- Keep the cooking utensils out of reach from children.
- Do not carry your child on the back when cooking.
- Install a gate to prevent children from entering the kitchen.
- Keep electrical appliances and cords out of reach from children.
- Install safety plugs on the electrical socket.
- Place the medicine in locked cupboard above waist level and keep them out of reach from the children.
- Consult doctors before feeding any Chinese Medicine or other medicine to children.
- Do not fill in water in the bathtub unattended.
- Install a safety gate in the washroom to keep the toilet and bathtub out of reach from children.
- Do not allow children to swim unsupervised and always accompany children when swimming.
- Do not take your child on board in a boat or ferry.

<u>9 months after birth</u>

- Parents' toys should be stored in a locked cabinet above waist level.
- Do not shake the child when playing.
- Do not stop the car suddenly and decelerate slowly.
- Do not put the child on the front seat when driving or put the child on a seat with a passenger air bag or hold the child when the car is moving.
- Install a car safety seat in the rear seat.
- Install finger pinch guards on the doors.
- Do not allow children to play with doors.
- Be careful when opening or closing doors.
- Install locks on cupboards.

<u>12 -18 months after birth</u>

- Put the medicine in a locked cabinet above waist level.
- Expired medicine should be disposed appropriately.
- Use safety caps on all bottles of medicine and keep the medicine out of reach from children.
- Check if there are damages of the painting and repair them.

- Lock and put the crib side up whenever your baby is in the crib.
- Do not leave your child unattended on bed, diaper changing mat, table or couch.
- Install window guards.
- Install gates around balconies.
- Use non-slip mat in bathtub and bathroom.
- Do not use walkers to avoid accidents.
- Fasten the seatbelt on the highchair or stroller.
- Do not allow your children to play in the bedroom or other places unattended.
- Parents should protect your children from direct sunlight. When going out, dress your children with thin long sleeves and sunglasses, use umbrella with UV protection and the Stroller cover.
- Use sunscreen for babies which protects from UVA and UVB. Apply on the back first for newly used sunscreen.
- Choose car safety seats according to the heights and weights of children and install the car safety seats in the rear seat. Always fasten the seat belt.
- Do not leave your child alone in the car and lock the car doors before driving.
- Do not allow children to play in or near the car.
