# Supplementary material for: Effectiveness of a Technology-Based Injury Prevention Program for Enhancing Mothers’ Knowledge of Child Safety: Protocol for a Randomized Controlled Trial
Source: JMIR Res Protoc. 2016 Oct 31;5(4):e205. doi: 10.2196/resprot.6216 (PMC5108924; doi:10.2196/resprot.6216)
Supplement: Multimedia Appendix 4 [file resprot_v5i4e205_app4.pdf]

The University of Hong Kong
香港大學兒童及青少年科學系
DEPARTMENT OF PAEDIATRICS AND ADOLESCENT MEDICINE
THE UNIVERSITY OF HONG KONG
Queen Mary Hospital
廣華醫院
Kwong Wah Hospital

Website and App User Acceptance Evaluation

Q1 – Usefulness Q2-Q4 Layout/structure/attractiveness Q5-Q8 Content - user friendliness Q9-Q10 Readability
Q11-Q12 Accessibility Q13-Q14 Satisfaction level

Please comment each of the following statements with the most appropriate answer:

1. Safety website and/or app is a useful website for caregiver (e.g. mother or father) to prevent child domestic injury.

Totally disagree	1	2	3	4	5	Totally agree

2. It is easy to find what you need from the safety website and/or app.

Totally disagree	1	2	3	4	5	Totally agree

3. Overall, The layout of the safety website and/or app is……………….

Extremely nebulous	1	2	3	4	5	Extremely clear

4. The appearance of the safety website and/or app is……………….

Visually unattractive	1	2	3	4	5	Visually appealing

5. The reading level of materials provided in the safety website and/or app is…………..

Extremely inappropriate for caregiver	1	2	3	4	5	Extremely appropriate for caregiver

6. The wording used in the safety website and/or app is……………….

Extremely difficult to understand	1	2	3	4	5	Extremely easy to understand

7. The information presented in the safety website and/or app is……………….

Totally vague	1	2	3	4	5	Totally understandable
Totally boring	1	2	3	4	5	Totally interesting
Totally incredible	1	2	3	4	5	Totally credible
One way communication	1	2	3	4	5	Interactive
Totally irrelevant to my needs	1	2	3	4	5	Totally relevant to my needs
Extremely inadequate	1	2	3	4	5	Extremely adequate

8. All the useful links provided are ………

Totally useless 1 2 3 4 5 Totally useful

9. It is difficult to move from page to page in the safety website and/or app.

Totally disagree 1 2 3 4 5 Totally agree

10. I cannot easily get back to the home page in the safety website and/or app.

Total disagree 1 2 3 4 5 Totally agree

11. I can easily search information in the safety website and/or app.

Totally disagree 1 2 3 4 5 Totally agree

12. My Internet browser can effectively access the safety website and/or app.

Totally disagree 1 2 3 4 5 Totally agree

13. I would recommend the safety website and/or app to others.

Totally disagree 1 2 3 4 5 Totally agree

14. Overall, my satisfaction level of the safety website and/or app is………

Extremely low 1 2 3 4 5 Extremely high
