# Supplementary material for: Effectiveness of a Technology-Based Injury Prevention Program for Enhancing Mothers’ Knowledge of Child Safety: Protocol for a Randomized Controlled Trial
Source: JMIR Res Protoc. 2016 Oct 31;5(4):e205. doi: 10.2196/resprot.6216 (PMC5108924; doi:10.2196/resprot.6216)
Supplement: Multimedia Appendix 5 [file resprot_v5i4e205_app5.pdf]

Responses to Health Care and Promotion Fund
Promotion Sub-committee (PSC) comments

PSC2: Provide details in the proposed website, including content and framework.

A: *Injury prevention information will be embedded systematically into the Hong Kong Childhood Injury Prevention and Research Association (CIPRA) website (http://childinjury.org.hk/).The existing version of CIPRA website already possesses with some key injury prevention information for the public. It will be further revamped and enhanced to fulfill the proposed project needs and facilitate target users. The website server is under the Department of Paediatrics, the University of Hong Kong and this is a Linux Server with a regular backup system to prevent any data loss.*

Under the website framework, a new section named "Home Safety Tips" will be built. It will be sub-divided into four main subgroups according to various safety issues relevant to infants/children's age groups. There will be different important injury prevention messages and information under each subgroup (Figure 1).

Participants will be required to register as members and login to the website to view the information provided in CIPRA. With the login system, we would be able to record all the actions taken by each user, including login time, duration of website surfing, contents that they have read, etc. The captured data can be used for further analysis and also for the purpose of incentive reimbursement.

PSC3: The project requires continued professional support to answer the questions. Any arrangement to engage professional support?

A: *There is no special engagement to engage professional support. However, the PA himself is an injury expert and the website has been managed and coordinated by a group of concerned professionals and academics dedicated to the prevention and research of childhood injuries in Hong Kong. Hence, there would be continued professional support to sustain the proposed project.*

PSC4: Suggest disseminating the findings of the project to relevant organizations, such as NGOs and Hospital Authority.

A: *The findings of the project and an additional leaflet about the domestic safety website with address link and brief introductory information will be disseminated to relevant*

public, private, and non-governmental organizations that concern about children health, in particular those promoting childhood safety.

PSC5: A governance system to adequately monitor the disbursement of cash or cash vouchers to ensure accountability and traceability is required.

A: The funding would be governed and managed by the University of Hong Kong. For the disbursement of cash or cash vouchers, a governance system would be set up to monitor the process. The Principal Applicant would keep record of the disbursement of cash or cash vouchers, and upon completion of follow-up evaluations, parents would be given cash vouchers as incentive.

*T***wo** *lists of subjects entitled for incentive would be tabulated and kept as records with the following information:*

-Project title
-Name and signature of recipient
-Name and signature of team member handling the cash/ cash voucher
-Description of the cash / cash voucher
-Date of disbursement

These records would be kept confidential and could be accessed by only the Principal Applicant and designated research team members. One list would be given to the Finance Department of the University of Hong Kong and the other one would be kept as internal record by the research team.

Figure 1.Tree-diagram of the website content:

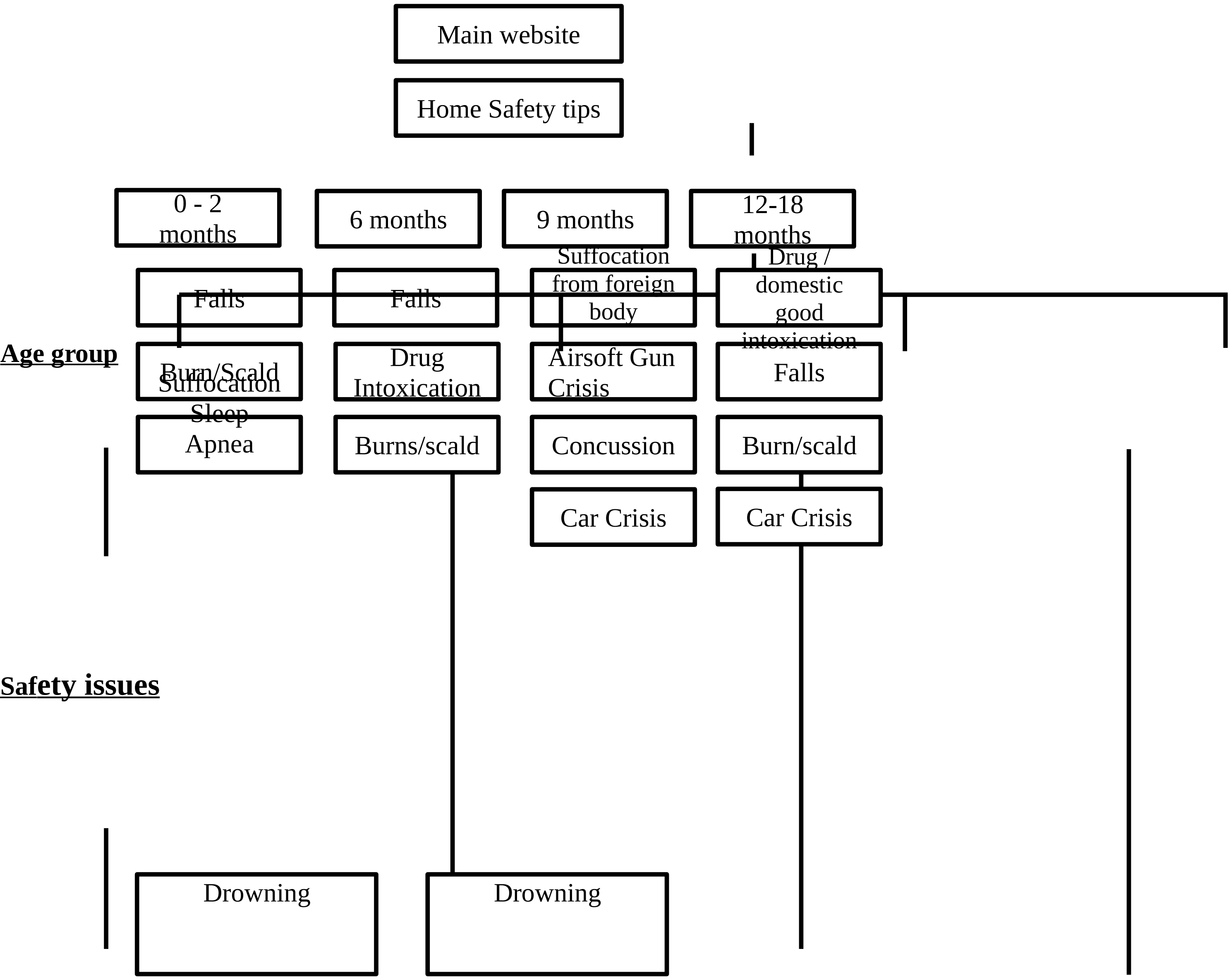

Environmental safety checklist: living room, bed room, bath room, kitchen…doors…corridor…
Domestic products safety checklist: bed, chair, table, sofa, toys, cabinets, TV set, baby walkers, clothing, widows, curtains, electric compliances, washing machine, refrigerator……
